# Synaptic and intrinsic plasticity within overlapping lateral amygdala ensembles following fear conditioning

**DOI:** 10.3389/fncel.2023.1221176

**Published:** 2023-10-09

**Authors:** Megha Sehgal, Vanessa E. Ehlers, James R. Moyer

**Affiliations:** ^1^Department of Psychology, University of Wisconsin-Milwaukee, Milwaukee, WI, United States; ^2^Department of Biological Sciences, University of Wisconsin-Milwaukee, Milwaukee, WI, United States

**Keywords:** intrinsic excitability, fear learning, lateral amygdala, synaptic plasticity, spike frequency adaptation

## Abstract

**Introduction:**

New learning results in modulation of intrinsic plasticity in the underlying brain regions. Such changes in intrinsic plasticity can influence allocation and encoding of future memories such that new memories encoded during the period of enhanced excitability are linked to the original memory. The temporal window during which the two memories interact depends upon the time course of intrinsic plasticity following new learning.

**Methods:**

Using the well-characterized lateral amygdala-dependent auditory fear conditioning as a behavioral paradigm, we investigated the time course of changes in intrinsic excitability within lateral amygdala neurons.

**Results:**

We found transient changes in the intrinsic excitability of amygdala neurons. Neuronal excitability was increased immediately following fear conditioning and persisted for up to 4 days post-learning but was back to naïve levels 10 days following fear conditioning. We also determined the relationship between learning-induced intrinsic and synaptic plasticity. Synaptic plasticity following fear conditioning was evident for up to 24 h but not 4 days later. Importantly, we demonstrated that the enhanced neuronal intrinsic excitability was evident in many of the same neurons that had undergone synaptic plasticity immediately following fear conditioning. Interestingly, such a correlation between synaptic and intrinsic plasticity following fear conditioning was no longer present 24 h post-learning.

**Discussion:**

These data demonstrate that intrinsic and synaptic changes following fear conditioning are transient and co-localized to the same neurons. Since intrinsic plasticity following fear conditioning is an important determinant for the allocation and consolidation of future amygdala-dependent memories, these findings establish a time course during which fear memories may influence each other.

## 1. Introduction

Amygdala plays a critical role in formation and storage of emotional memories (LeDoux, 2000). Emotionally maladaptive states like generalized anxiety disorder and post-traumatic stress disorder can result from aberrant amygdala plasticity (Davis, 1992; Mahan and Ressler, 2012). Given the prevalence of such disorders, a better understanding of the neural plasticity mechanisms that regulate allocation, consolidation, and integration of fear memories is imperative.

New learning is supported by plasticity in the underlying neuronal ensembles. While changes in the intrinsic neuronal excitability (i.e., intrinsic plasticity) as well as synaptic plasticity following learning are well documented (Maren and Baudry, 1995; Sehgal et al., 2013), their precise contributions to memory encoding are less clear (Lisman et al., 2018). The prevailing view is that synaptic plasticity is necessary for encoding and expression of memory (Ryan et al., 2015), whereas intrinsic plasticity mechanisms are critical for modulating the strength and allocation of memories during learning (Zhou et al., 2009; Yiu et al., 2014; Sehgal et al., 2018). Specifically, modulation of neuronal intrinsic excitability in the memory encoding neurons might underlie ongoing organization and linking of memories (Sehgal et al., 2013, 2018; Rogerson et al., 2014; Cai et al., 2016; Rashid et al., 2016).

We and others have demonstrated that new learning results in an increase in neuronal intrinsic excitability in the underlying brain regions (for example, Moyer et al., 1996; Saar et al., 1998; Song et al., 2012; Sehgal et al., 2013, 2014; Motanis et al., 2014). In hippocampal subregions, these learning-related changes in neuronal intrinsic excitability are transient (Moyer et al., 1996; Thompson et al., 1996). Experimental as well as behavioral manipulations of intrinsic excitability can bias neurons to be preferentially recruited into a memory (Han et al., 2007; Zhou et al., 2009; Yiu et al., 2014; Cai et al., 2016; Rashid et al., 2016). Specifically, memory formation results in a period of enhanced intrinsic excitability in a subset of neurons, and neurons with higher intrinsic excitability are preferentially recruited to encode future memories (Cai et al., 2016; Rashid et al., 2016; Sehgal et al., 2018; Shen et al., 2022). This allows for overlap between the neuronal ensemble encoding two temporally proximate memories such that these memories are behaviorally linked (Cai et al., 2016; Rashid et al., 2016).

Since learning-related intrinsic plasticity can impact memory organization for a period, the time course of these changes is important to understand memory organization in these brain regions. We have previously demonstrated that fear learning results in increased neuronal excitability in a subset of lateral amygdala (LA) neurons (Sehgal et al., 2014) but the time course of these changes is unclear. In the current study, we determine the time course of intrinsic plasticity within lateral amygdala neurons following fear conditioning. In addition, we use synaptic plasticity measurements from the same neurons to establish whether these neurons were responsible for encoding the fear memory within the lateral amygdala. Our data suggest that fear conditioning results in long-lasting but transient changes in the intrinsic excitability of lateral amygdala neurons (up to 4 days). Importantly, these changes in lateral amygdala neuronal excitability are correlated with synaptic facilitation in these neurons but only immediately following fear conditioning.

## 2. Results

Using sharp intracellular recordings, we previously demonstrated that fear conditioning results in enhanced intrinsic excitability within a subset of lateral amygdala neurons. These changes were still present up to 24-h following fear conditioning, which was the longest time point tested in that study (Sehgal et al., 2014). To determine the time course of intrinsic excitability changes in the lateral amygdala following fear conditioning, we obtained electrophysiological recordings from LA neurons from animals that were fear conditioned 1 h, 1 day, 4 days, or 10 days ago.

### 2.1. Fear conditioning results in robust and long-lasting increase in freezing behavior

**Figure 1:**
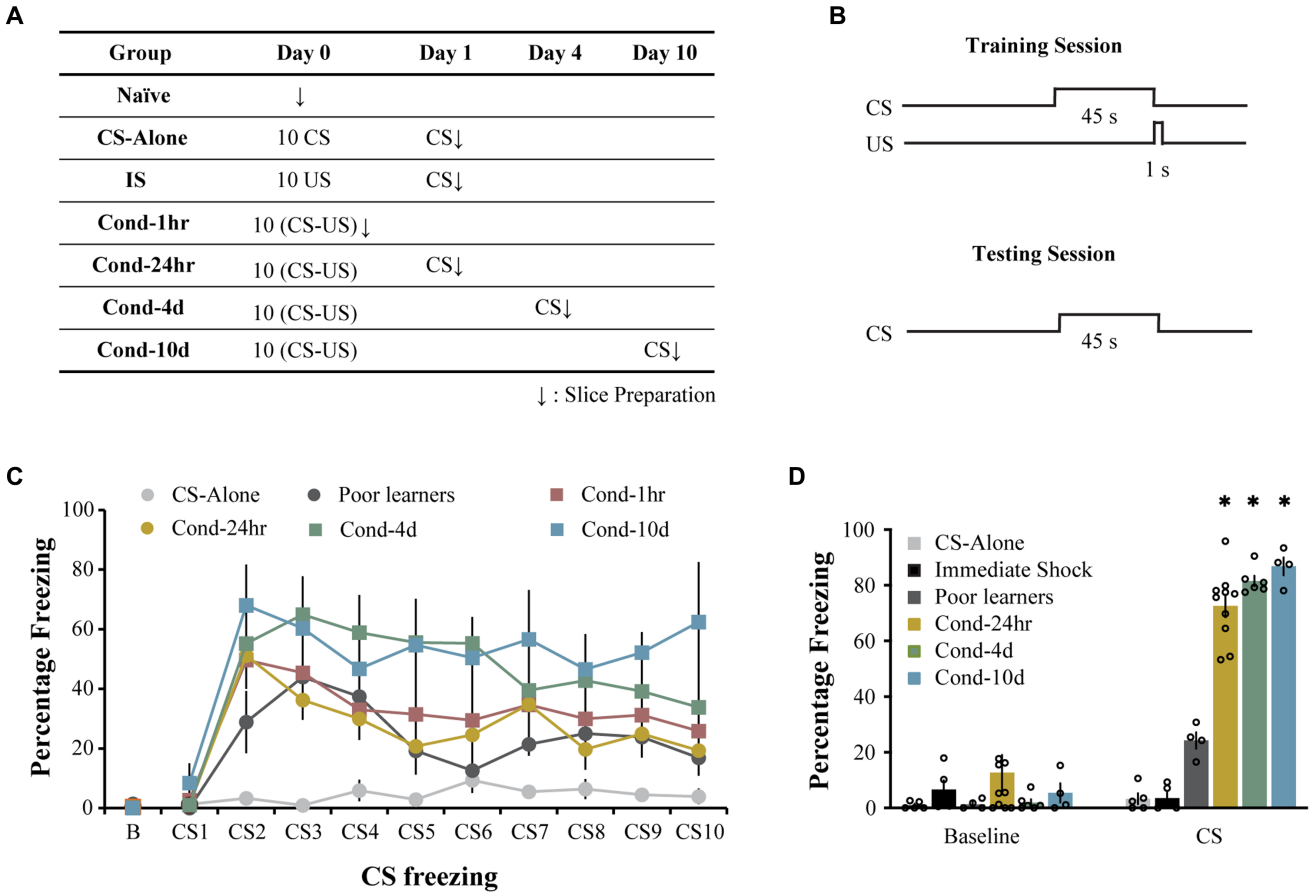
Long-delay fear conditioning is readily acquired and lasts up to 10 days. **(A)** Experimental design used to study intrinsic plasticity in the lateral amygdala neurons following fear conditioning. Behavioral groups. Rats were divided into seven groups: three control groups [naïve, CS-alone and Immediate Shock (IS)] and four experimental groups [Cond-1 h, Cond-24 h, Cond-4d, and Cond-10d]. **(B)** Rats were trained using a long-delay fear conditioning protocol (CS: 45 s, US: 1 s, 1 mA footshock, 5.2 min ITI). **(C)** Training. During the fear conditioning session on day 1, CS-alone rats (*N* = 5) froze significantly less than conditioned rats in Cond-1 h (*N* = 8), Cond-24 h (*N* = 10), Cond-4d (*N* = 6), and Cond-10d (*N* = 4) rats. **(D)** Testing. During the probe test, the IS rats displayed low levels of freezing to the training context (not significantly different from the baseline freezing for all other groups in a novel context). During CS presentation, rats in the conditioned groups (Cond-24 h, Cond-4d, and Cond-10d) displayed significantly higher freezing than CS-alone, IS and Poor learners. Asterisk (*) indicates *p* < 0.05 relative to the Poor learners group. CS, freezing during CS presentation; IS, immediate shock.

Rats were trained on a lateral amygdala-dependent auditory fear conditioning paradigm as previously described (Sehgal et al., 2014). Following auditory fear learning, all animals (other than the immediate shock group) were tested using a CS presentation in a novel context to assess the strength of auditory fear memory. The immediate shock (IS) group was tested in the original training context to assess the lack of context-US association. Electrophysiological recordings were made immediately following the recall test (Figures 1A,B). During the conditioning session, rats from all groups displayed low levels of baseline freezing (0.41 ± 0.14%) with no significant differences observed between groups [one way ANOVA, *F* (5, 31) = 1.8, *p* = 0.13; Figure 1C]. Following the baseline period, rats in all the conditioning groups displayed comparable levels of increased freezing while the control group, CS-alone, that received CS presentations in the absence of the US displayed low levels of freezing (repeated measures ANOVA, significant effect of training trial [*F*(5.9, 181.4) = 16.2, *p* < 0.001; Greenhouse-Geisser corrected] as well as group [*F*(5, 31) =4.7, *p* < 0.005], Figure 1C). Pairwise comparisons demonstrated that rats in the CS-alone group froze less during CS presentations than rats in all other groups except the Poor learners (all *p* values < 0.05). While all other groups displayed higher freezing with the training trial (*p* < 0.05), the CS-alone group did not display a significant increase in CS freezing over the course of conditioning. These data indicate that all animals that received paired presentations of the CS and US displayed successful acquisition of fear learning relative to control animals receiving CS presentations alone.

Similarly, during the testing session, the average baseline freezing was low across all groups (6.0 ± 2.1%, Figure 1D) and there were no significant differences between the groups [One way ANOVA, *F* (5, 30) = 1.07, *p* = 0.40]. Specifically, the immediate shock (IS) group, which was tested in the original training context to assess for the lack of context-US association, also displayed low baseline or context freezing (6.6 ± 3.32%). Therefore, as expected, the IS rats did not form a fearful association between the training context and the US presentation and are thus an appropriate control for the footshock presentation in the absence of learning.

Following the baseline period, rats in the fear conditioned groups (i.e., Cond 24 h, Cond 4d, and Cond 10d) displayed robust freezing to the CS presentation. Some rats displayed low levels of fear to the CS presentation (less than 50% freezing) and were separated into a Poor learner group. A one-way ANOVA revealed a significant effect of group on CS freezing [*F*(5, 28) = 117.7, *p* < 0.001]. *Post hoc* tests indicated that the control groups (CS-alone and immediate shock) as well as Poor learners displayed significantly lower CS freezing than all the conditioned groups (*p* < 0.001) with no significant differences within control or conditioned groups. Thus, the rats in the conditioned groups displayed robust fear memory up to 10 days following fear conditioning while the rats exposed to CS or US presentations alone displayed no fear learning.

### 2.2. Fear conditioning transiently enhances intrinsic excitability

**Figure 2:**
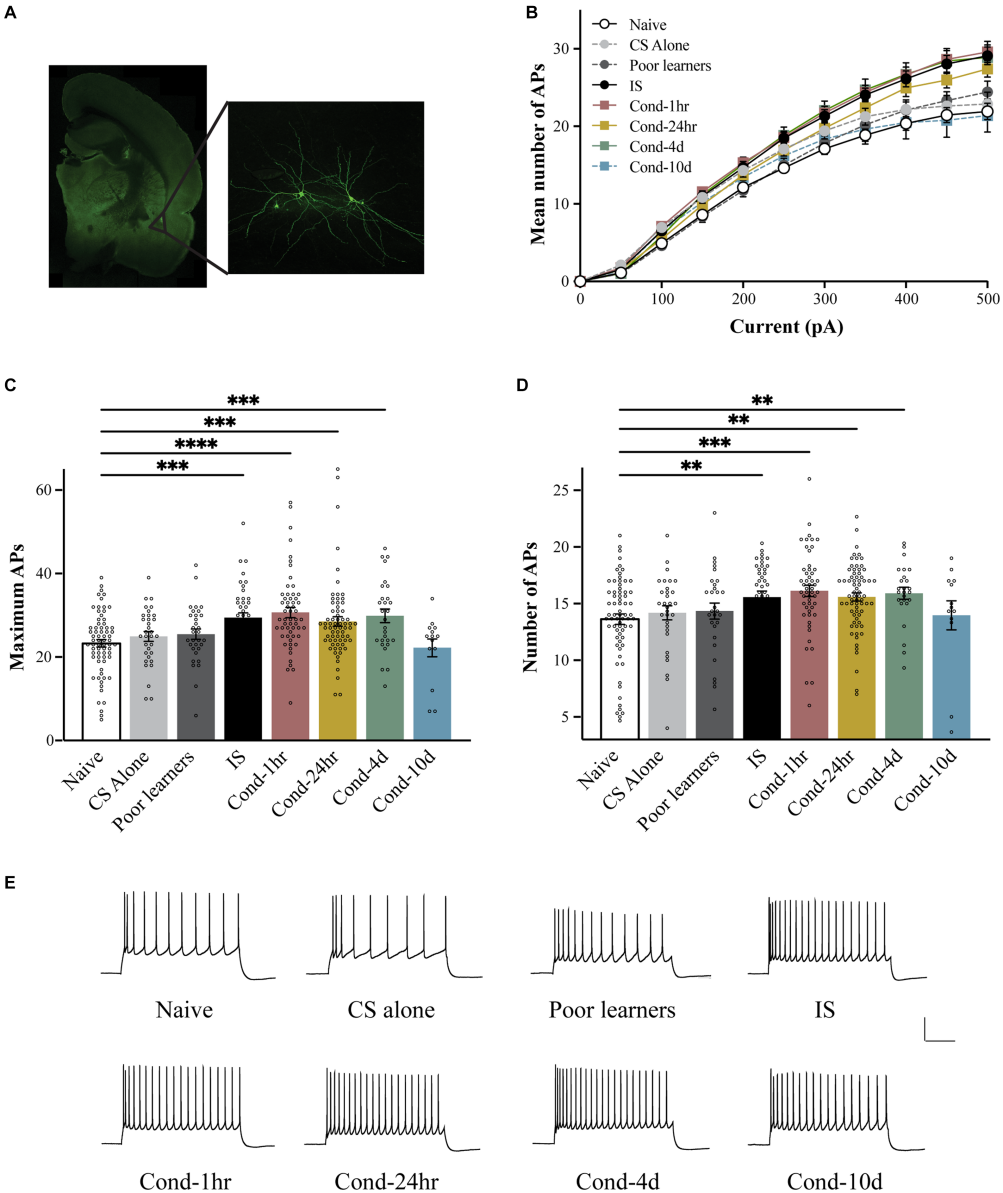
Fear conditioning results in a transient increase in the intrinsic excitability of LA neurons. **(A)** Following behavioral training, lateral amygdala containing coronal slices were made and patch clamp electrophysiology recordings were performed. *Left panel* is a photomicrograph of a typical coronal brain slice showing the location of the lateral amygdala (LA). *Right panel* shows a pair of biocytin-filled LA pyramidal neurons. **(B)** LA neurons from Cond-1 h, Cond-24 h, Cond-4d, and IS group rats fired significantly more action potential than those from naïve rats (300–500 pA; *p* < 0.05). LA neurons from CS-alone, Poor learners and Cond-10d group did not differ from LA neurons from naïve rats. **(C)** Maximum number of action potentials fired during current injections ranging from 0 to 500 pA were significantly increased in LA neurons from Cond-1 h, Cond-24 h, Cond-4d, and IS group rats but not for LA neurons from CS-alone, Poor learners and Cond-10d group. **(D)** Fear conditioning reduces spike frequency adaptation within the amygdala. LA neurons from Cond-1 h, Cond-24 h, Cond-4d, and IS group rats fired significantly more action potentials than those from naïve rats (300–500 pA; *p* < 0.05). No significant differences were found among naïve, CS-alone, Poor learners and Cond-10d group. **(E)** Representative voltage sweeps of action potentials fired following a 300 pA somatic current injection for LA neurons from the various behavioral groups. Scale: 30 mV, 0.25 s. Asterisk (*) indicates *p* < 0.05. CS, conditioned stimulus; IS, immediate shock.

To determine the time course of intrinsic excitability changes following fear conditioning, we used patch clamp whole-cell recordings to assess LA neuronal excitability. LA neurons were injected with 1 s long current pulses ranging from 0 to 500 pA and the number of action potentials (APs) elicited during the pulse were counted (Figure 2). A repeated-measures ANOVA indicated a significant effect of current [*F*(1.83, 606.7) = 1984.4, *p* < 0.001; Greenhouse-Geisser corrected], behavioral condition [*F*(7, 332) = 5.3, *p* < 0.001] as well as a group by current interaction [*F*(12.8, 606.7) = 5.589, *p* < 0.001; Greenhouse-Geisser corrected] on the number of APs elicited. *Post hoc* analyses revealed that LA neurons from Cond-1 h, Cond-24 h, Cond-4d as well as the IS group fired significantly more APs relative to neurons from naïve rats (*p* < 0.05). In addition, the number of APs elicited did not differ between naïve, CS-alone, Poor learners, and Cond-10d groups. Follow-up one-way ANOVAs indicated a significant effect of behavioral condition on the number of APs elicited for current injections ranging from 100 to 500 pA (all *p* values < 0.05). *Post hoc* comparisons confirmed that relative to LA neurons from naïve rats, LA neurons from Cond-1 h, Cond-24 h, Cond-4d and IS group fired more APs following a 300–500 pA current injection. Furthermore, *post hoc* comparisons also confirmed that relative to LA neurons from CS alone rats, LA neurons from Cond-1 h, Cond-4d and IS groups fired more APs following a 450–500 pA current injection (all *p* values < 0.05; for Cond-24 h, *p* = 0.09 and *p* = 0.03 for injections of 450 pA and 500 pA respectively). Finally, LA neurons from Cond-10d rats had excitability measures that were similar to naïve animals (all *p* values > 0.05) but significantly lower than other conditioned animals (Cond-1 h, Cond-24 h, and Cond-4d, all *p* values < 0.05) indicating that intrinsic excitability was back to naïve values 10 days later. These data demonstrate that successful acquisition of fear conditioning leads to a transient increase in LA neuronal excitability that returns to naïve levels by 10 days following fear conditioning. These changes in excitability were not observed in LA neurons from rats that failed to acquire fear conditioning (i.e., Poor learners). However, LA neurons from rats in the IS group also displayed increased intrinsic excitability indicating that shock presentation alone could lead to alterations in LA intrinsic excitability.

Although the neuronal response to a stepwise increase in somatic depolarization is widely used as a measure of neuronal excitability, subtle changes in input resistance, and in certain cases the cessation of AP firing following very high somatic depolarizations, can influence this measure of excitability. To obtain additional indices of intrinsic excitability, we also measured the maximum number of APs fired during current injections ranging from 0 to 500 pA (Figure 2C) and spike frequency adaptation (Figure 2D). Once more, a one-way ANOVA revealed a significant effect of behavioral group on the maximum number of APs elicited [*F*(7, 335) = 6.95, *p* < 0.001]. *Post hoc* tests confirmed that relative to LA neurons from naïve rats, those from IS, Cond-1 h. Cond-24 h and Cond-4d rats exhibited an increase in the maximum number of APs elicited (all *p* values < 0.05). No significant differences were found between the naïve and other control groups (i.e., CS-alone and Poor learners) as well as the Cond-10d group. Similar to our previous findings (Figure 2B), *post hoc* tests confirmed that LA neurons from IS, Cond-1 h, Cond-24 h and Cond-4d rats had an increased number of maximum APs (all *p* values < 0.05) relative to those from CS-alone group. These data confirm our earlier observation of a transient increase in intrinsic excitability within LA neurons of conditioned animals. In addition, we also demonstrated an increase in the intrinsic excitability of LA neurons from IS animals in the absence of learning.

To obtain a measure of spike frequency adaptation, a current injection sufficient to elicit 3 APs in the first 100-ms was injected over a period of 1 s and the number of APs elicited were counted (Figure 1D). Again, a one-way ANOVA demonstrated a significant effect of behavioral condition on the number of APs elicited [*F*(7, 321) = 3.5, *p* < 0.001]. *Post hoc* tests confirmed that LA neurons from Cond-1 h, Cond-24 h, Cond-4d and IS rats fired significantly more APs relative to LA neurons from naïve rats (all *p* values < 0.05). As before, no significant differences were found between the number of APs elicited in LA neurons from Poor learners, CS-alone and Cond-10d relative to naïve rats. These data demonstrate that (1) rats that successfully acquire fear conditioning display an increase in LA neuronal excitability relative to rats that do not learn, (2) these intrinsic excitability changes are transient, lasting up to 4 days before returning back to naïve levels by 10 days, and (3) LA neuronal intrinsic excitability may also increase following footshock US-alone presentations.

### 2.3. Passive membrane and action potential properties

Changes in neuronal excitability can also result from changes in certain passive membrane properties (Table 1). We controlled for any effect of variations in resting membrane potential (RMP) by obtaining all our intrinsic excitability measurements at −60 mV. In addition, we did not observe any significant differences in RMP between groups [*F*(7, 336) = 1.0, *p* = 0.4]. Input resistance of the neuron can also affect the resulting depolarization following a somatic current injection. Like RMP, input resistance was also unchanged between various groups [*F*(7, 336) = 0.367, *p* = 0.9]. These data indicate that any changes in the intrinsic excitability of LA neurons observed following behavioral training did not result from changes in passive membrane properties. Furthermore, since changes in action potential properties can also accompany changes in the intrinsic excitability of a neuron, we measured single AP properties and found no significant differences in AP amplitude [*F*(7, 305) = 0.583, *p* = 0.8] or AP halfwidth among the behavioral groups [*F*(7, 305) = 0.502, *p* = 0.8]. Therefore, fear conditioning did not alter action potential amplitude or duration.

**Table 1:** Effects of fear conditioning on basic membrane and AP properties of LA neurons.

Group	*n* (*N*)	RMP (mV)	*R_N_* (MΩ)	AP properties	
				Amplitude (mV)	Halfwidth (ms)
Naive	68 (14)	−61.5 ± 0.7	221.5 ± 6.7	66.8 ± 1.2	1.1 ± 0.02
CS-alone	33 (5)	−62.1 ± 0.8	217.4 ± 10.9	69.6 ± 1.5	1.2 ± 0.02
Poor learners	32 (4)	−61.7 ± 1.06	219.5 ± 15.8	68.4 ± 1.9	1.1 ± 0.04
IS	42 (5)	−59.7 ± 0.9	210.6 ± 10.3	69.2 ± 1.2	1.3 ± 0.33
Cond-1 h	58 (9)	−60.4 ± 0.9	214.9 ± 8.7	66.7 ± 1.6	1.1 ± 0.02
Cond-24 h	70 (10)	−60.3 ± 0.7	205.6 ± 7.5	67.3 ± 1.0	1.1 ± 0.02
Cond-4d	26 (6)	−59.6 ± 1.2	200.6 ± 9.7	67.3 ± 1.7	1.1 ± 0.03
Cond-10d	14 (4)	−62.8 ± 0.9	219.9 ± 15.0	68.6 ± 2.0	1.2 ± 0.08

Data are presented as the mean ± SE. AP properties were measured from threshold. *n*, number of cells; *N*, number of animals; RMP, resting membrane potential; *R_N_*, input resistance; AP, action potential; IS, immediate shock.

### 2.4. Synaptic plasticity following fear conditioning

To investigate the relationship between intrinsic and synaptic plasticity following fear conditioning, we obtained measurements of synaptic strength as well as intrinsic excitability from the same neurons. Rats were fear conditioned as described before and control groups included naïve, CS-alone, or IS rats. After obtaining intrinsic excitability measurements under current clamp mode, neurons were voltage clamped at −70 mV and measures of synaptic strength were obtained.

**Figure 3:**
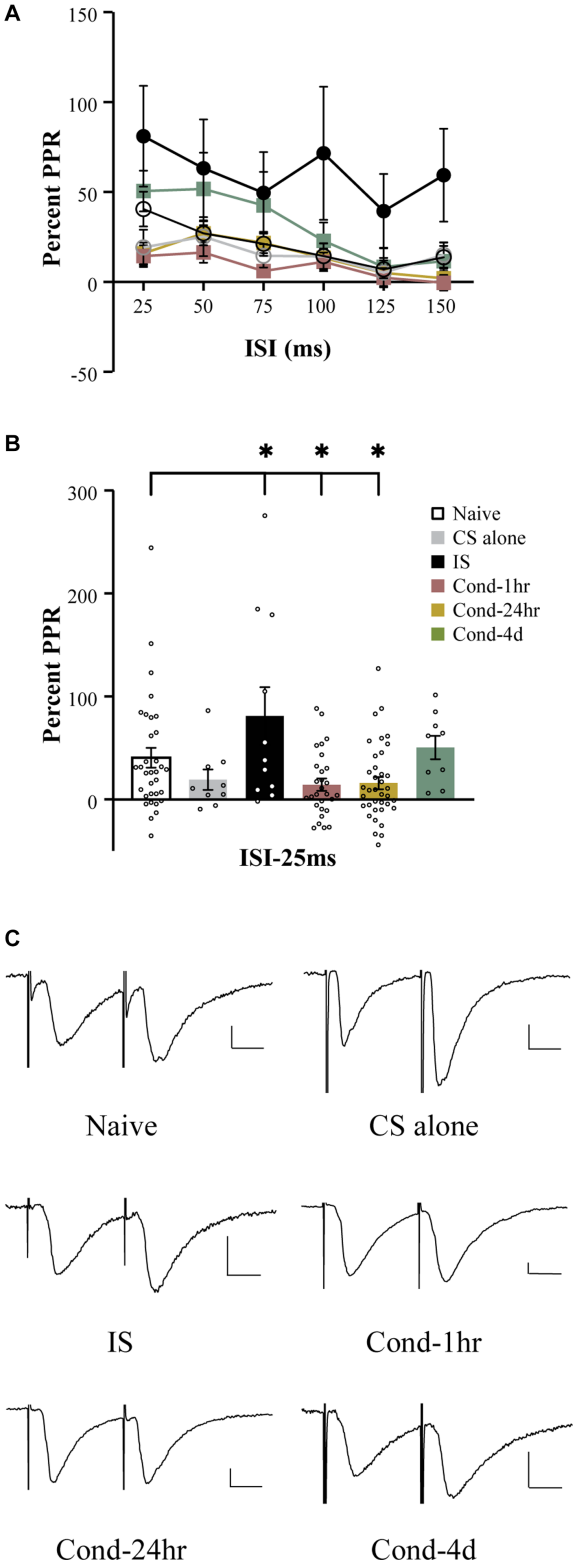
Fear conditioning reduces paired pulse facilitation within thalamo-amygdala synapses. **(A)** Line graph representing the percent PPR as a function of ISI (25–150 ms) and behavioral condition. The PPR is reduced as the ISI between the presynaptic stimuli is increased. The PPR for LA neurons from IS group is larger relative to LA neurons from naïve rats at all ISIs tested. **(B)** LA neurons from Cond-1 h and Cond-24 h display significantly lower PPR at 25 ms ISI relative to naïve LA neurons. LA neurons from CS-alone and Cond-4d were not different from naïve LA neurons. **(C)** Representative current sweeps displaying changes in PPR of LA neurons. Scale 25 pA, 10 ms. Asterisk (*) indicates *p* < 0.05 relative to LA neurons from naïve rats. PPR, paired pulse ratio; CS, conditioned stimulus; IS, immediate shock.

Fear learning results in synaptic plasticity within amygdala neurons (McKernan and Shinnick-Gallagher, 1997; Clem and Huganir, 2010) and a well-established measure of synaptic plasticity is the paired pulse ratio (PPR). To measure the PPR, pairs of EPSCs were generated by temporally close presynaptic stimulation of thalamic fibers synapsing onto the LA neurons. Stimulation intensity was adjusted to obtain a reliable EPSC ranging from 50 to 150 pA. Baseline measurements were obtained for 1 min to establish that the EPSC amplitude remained stable. The interstimulus interval varied from 25 ms to 150 ms (Figure 3A). A repeated measures ANOVA revealed a significant effect of interstimulus interval [*F*(4.0, 460.9) = 13.9, *p* < 0.001; Greenhouse-Geisser corrected], group [*F*(5, 116) = 3.62, *p* < 0.005] as well as a group by time interaction [*F*(19.9, 460.9) = 1.65, *p* < 0.05; Greenhouse-Geisser corrected]. A follow-up one-way ANOVA demonstrated a significant effect of behavioral training on PPR at the shortest interstimulus interval (ISI), i.e., 25 ms [*F*(5,121) = 4.5, *p* < 0.001]. *Post hoc* tests revealed that while LA neurons from naïve rats displayed enhancement of the second EPSC (i.e., paired pulse facilitation), this facilitation was significantly attenuated in LA neurons from the Cond-1 h as well as Cond-24 h rats (Figure 3B, *p* < 0.05). Interestingly, IS rats were also significantly different from the naïve rats but in the opposite direction than that of conditioned animals (*p* < 0.05). In other words, the PPR was significantly *higher* for LA neurons from IS rats compared to those from naïve rats. No significant differences were observed between naïve, CS-alone and Cond-4d rats. These data as well as various other published reports (e.g., McKernan and Shinnick-Gallagher, 1997) demonstrate that fear learning is accompanied by a reduction in paired pulse ratio, a well-accepted measure of synaptic plasticity.

In addition to a change in paired pulse ratio at a short ISI of 25-ms, follow up ANOVAs demonstrated a significant effect or strong trend of behavioral group on PPR at various other time points (all *p* values < 0.08). *Post hoc* tests revealed that these effects were driven by a significant increase in PPR in LA neurons from IS rats relative to those from naïve rats (*p* < 0.05). Interestingly, in contrast to all other behavioral groups varying the interstimulus interval between the pairs of EPSCs had no significant effect on the PPR for the IS group [repeated measure; *F* (5, 50) = 1.822, *p* = 0.13]. Therefore, the synaptic plasticity observed in the IS group is more robust and in the opposite direction to that observed in the conditioned animals.

### 2.5. Correlation between synaptic and intrinsic plasticity

**Figure 4:**
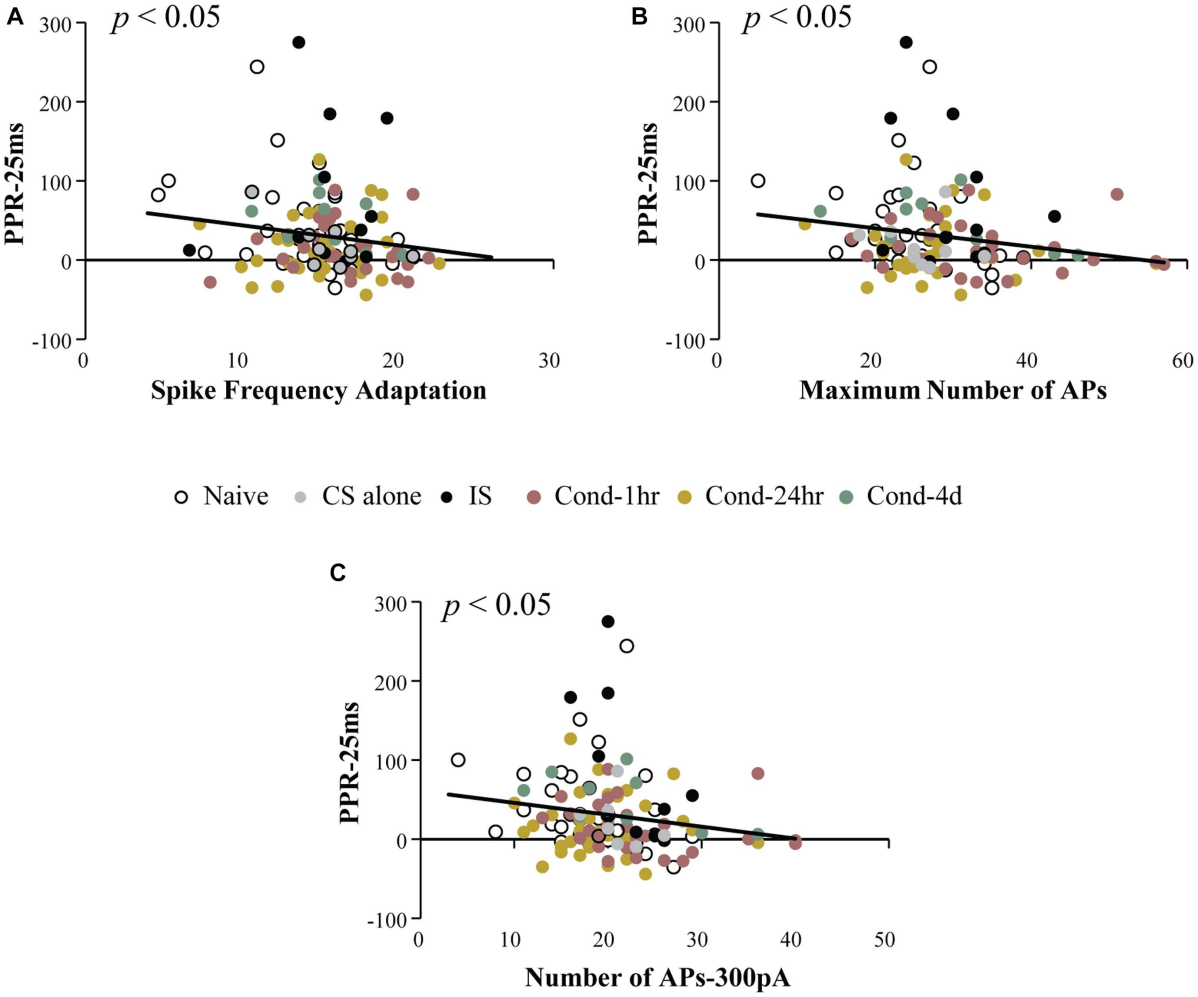
Intrinsic excitability measures are correlated with paired pulse ratio. PPR at 25 ms ISI was plotted as a function of intrinsic excitability measures. Spike frequency adaptation **(A)**, maximum number of action potentials fired for 0–500 pA current injection **(B)** and number of action potentials fired following a 300 pA current injection **(C)** are significantly correlated with PPR (*p* < 0.05). Asterisk (*) indicates *p* < 0.05. AP, action potential; PPR, paired pulse ratio.

The primary aim of this analysis was to investigate whether intrinsic and synaptic plasticity were co-localized within the same neurons following fear conditioning. To determine this, we measured the relationship between PPR at a 25-ms ISI (PPR-25) and various measures of intrinsic excitability. Notably, PPR-25 was found to be modestly but significantly correlated with spike frequency adaptation, the number of APs elicited following current injections ranging from 50 to 500 pA, as well as maximum number of APs elicited (Spearman’s correlations = −0.181, −0.231 (300 pA), and −0.217 respectively; all *p* values < 0.05, Figure 4). This correlation was not dependent on the behavioral group and was also observable in LA neurons from naïve animals (Spearman’s correlation = −0.36, *p* < 0.05). Thus, a significant correlation exists between synaptic efficacy and intrinsic excitability which we analyzed more thoroughly below.

**Figure 5:**
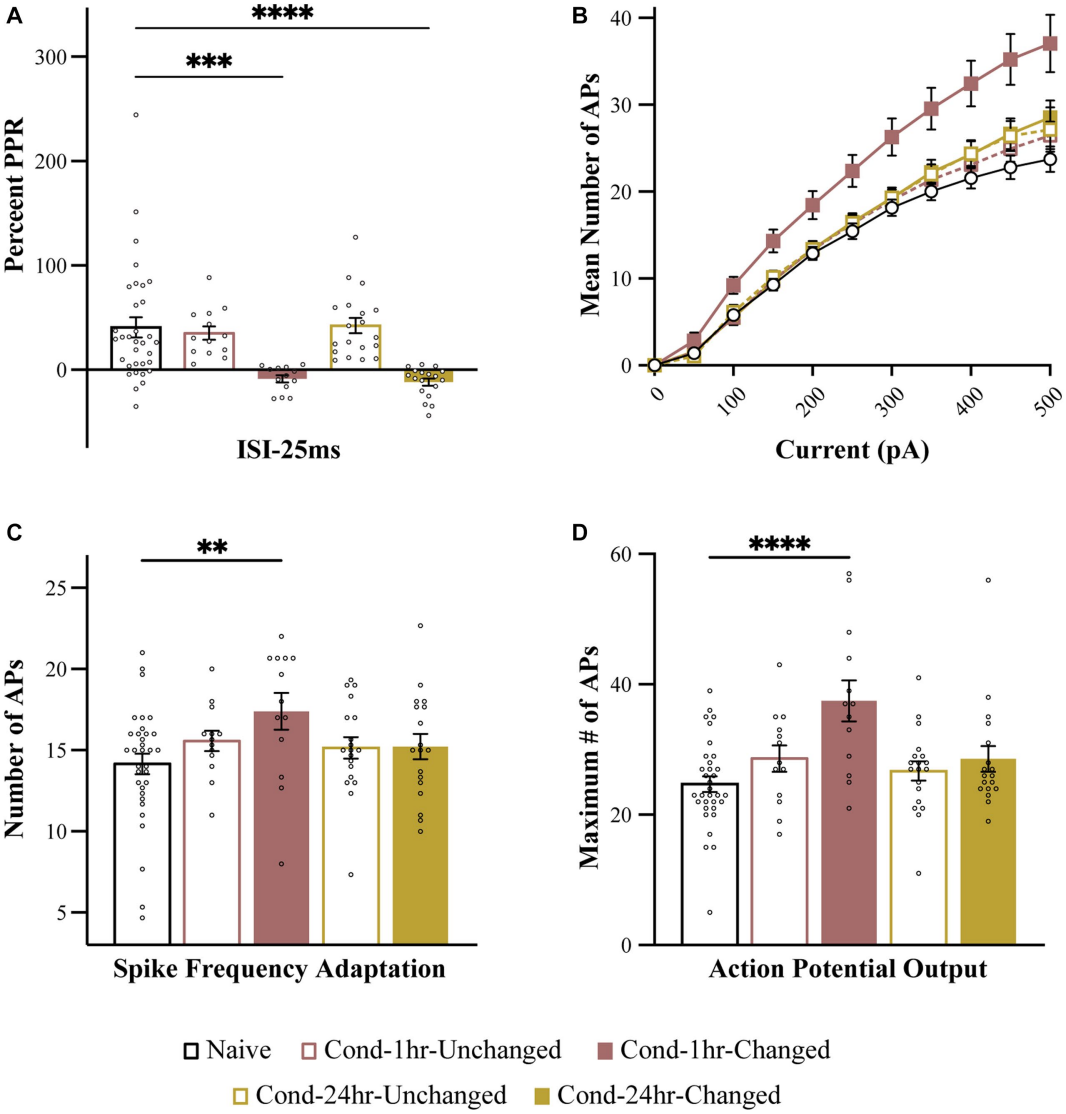
Intrinsic excitability changes are colocalized to the neurons undergoing synaptic plasticity immediately following fear conditioning. **(A)** PPR is significantly reduced in Cond-1 h changed and Cond-24 h changed neurons relative to naive as well as Cond-1 h unchanged and Cond-24 h unchanged groups. **(B)** Number of APs elicited following 0–500 pA current injection are increased in Cond-1 h changed group neurons relative to other groups. **(C)** Spike frequency adaptation is reduced in Cond-1 h changed but not in any other group relative to naïve (*p* < 0.05). **(D)** Maximum number of action potentials are also significantly increased in Cond-1 h changed group relative to others. Asterisk (*) indicates *p* < 0.05 relative to Naive. AP, action potential; PPR, paired pulse ratio.

To further analyze the relationship between these two forms of plasticity, we split the neurons from Cond-1 h and Cond-24 h group into high (unchanged) and low (changed) PPR based on the median PPR-25 value for these groups (Figure 5). This is especially important as correlations are highly sensitive to outliers and non-normal distribution of data, and thus a median split allows us to better analyze the presence or absence of co-localization of these two forms of plasticity. The neurons in the low PPR or changed groups should be the ones that display reduced PPR because of fear learning.

As predicted, a one-way ANOVA revealed a significant effect of group on PPR [*F* (4, 91) = 9.4, *p* < 0.001]. The PPR was significantly reduced for Cond-1 h changed as well as Cond-24 h changed neurons but not the Cond-1 h unchanged or the Cond-24 h unchanged neurons (*p* < 0.001). More importantly, we next analyzed the measures of intrinsic excitability based on these new groups. One-way ANOVA revealed that the number of APs elicited following a series of current pulses ranging from 0 to 500 pA were significantly different between the groups. This effect emerged at current injections of 100 pA and remained significant up to 500 pA (all *p* values < 0.05). Interestingly, these results were driven by a significant increase in AP firing in Cond-1 h changed neurons [*F*(4, 91) = 6.4, *p* < 0.001] relative to neurons from the naïve group. No significant differences were found between Cond-1 h unchanged, Cond-24 h changed, Cond-24 h unchanged and naïve groups. Relative to neurons from the naïve group, the maximum number of APs elicited during these current steps were also significantly increased for Cond-1 h changed but not any of the other groups (*p* < 0.001). A one-way ANOVA revealed a strong trend for a similar effect on spike frequency adaptation [*F*(4, 91) = 2.26, *p* = 0.07]. *Post hoc* tests confirmed that LA neurons from Cond-1 h changed group were more excitable relative to neurons from the other groups (*p* < 0.005). These data demonstrate that within LA neurons, potentiation of intrinsic excitability is co-localized with synaptic plasticity, immediately but not 24 h following fear conditioning.

We further substantiated this analysis by classifying neurons as “*changed*” when the PPR-25 was two standard deviations below the mean Naïve PPR-25. This method of detecting changed neurons resulted in similar conclusions where PPR was reduced for Cond-1 h changed as well as Cond-24 h changed neurons but not the Cond-1 h unchanged or the Cond-24 h unchanged neurons [*F*(4, 91) = 11.0, *p* < 0.001, *post hoc p* < 0.001]. Measures of intrinsic excitability were only increased in Cond-1 h changed group but not in other groups [Number of spikes for current injections: *F*_interaction_ (40, 910) = 4.1, for 100–400 pA all *p* values < 0.05; Maximum APs: *F*(4, 91) = 5.6, *p* < 0.001]; Adaptation: [*F*(4, 91) = 2.8, *p* < 0.05, all *post hoc p* values < 0.05]. Therefore, our conclusion that potentiation of intrinsic excitability, as well as synaptic efficacy, is co-localized immediately following fear conditioning is robust and does not depend on our method of analysis.

## 3. Discussion

For the current study, we sought to determine the time course of intrinsic plasticity following auditory fear conditioning within the lateral amygdala. We found that intrinsic excitability is increased immediately, and up to 4 days following fear conditioning. These changes are transient as the increase in excitability is no longer evident at 10 days following fear conditioning even though fear memory retrieval is unaffected. The increase in intrinsic excitability was also evident in the immediate shock group which received shock presentations for the same amount of time as the conditioned animals but displayed no evidence of learning the day after. Since conditioned animals that failed to acquire fear conditioning did not display intrinsic plasticity, we conclude that fear conditioning as well as footshock US presentations can enhance the intrinsic excitability of lateral amygdala neurons.

### 3.1. Intrinsic plasticity is transient

As previously described, a majority of studies investigating the time course of intrinsic plasticity demonstrate that it is transient, lasting less than 7–14 days (Moyer et al., 1996; Saar et al., 1998; Zelcer et al., 2006; Motanis et al., 2014). However, long lasting changes in the intrinsic excitability of cerebellar Purkinje neurons following eyeblink conditioning can last for up to 1 month (Schreurs et al., 1998). Therefore, it is possible that the persistence of learning-related intrinsic plasticity is different between structures such as the hippocampus and the amygdala especially given their differential role in memory processes over the longer term (Kim and Fanselow, 1992; Do-Monte et al., 2015).

Here we found that changes in LA neuronal excitability following fear conditioning were transient and were back to naïve levels by 10 days following fear conditioning. This is in accordance with data from olfactory discrimination learning studies where changes in neuronal excitability within basolateral amygdala started to reverse by 3 days following learning (Motanis et al., 2014). Thus, intrinsic plasticity following behavioral training within the amygdala appears to be transient. The transient nature of such learning-related plasticity can be informative. It follows that any effect of amygdala intrinsic plasticity on future plasticity, synaptic or behavioral is likely to be transient as well. Indeed, transient increases in intrinsic excitability can impact memory organization and linking via such time-dependent effects (Sehgal et al., 2013, 2018; Cai et al., 2016; Rashid et al., 2016; Lisman et al., 2018).

Retrieval of a context or contextual fear memory can result in a transient increase of neuronal excitability in dentate gyrus neurons (Pignatelli et al., 2019). Since a majority of conditioned animals (i.e., Cond-24 h, Cond-4d, and Cond-10d) in our study also received a retrieval session immediately before slice preparation, it is possible that the enhanced excitability in these groups can result from conditioning or the subsequent retrieval session. Incorporation of a retrieval session is an experimental design that is commonly employed to establish that changes in excitability do not reflect changes in memory recall and allows for the correlation of behavioral performance with observed intrinsic plasticity (for example, Moyer et al., 1996; Cohen-Matsliah et al., 2009; Kaczorowski and Disterhoft, 2009; Song et al., 2012, 2015; Sehgal et al., 2014). We previously demonstrated that LA neurons display increased intrinsic excitability 24-h post-fear conditioning with and without a retrieval session (Sehgal et al., 2014). Therefore, fear conditioning itself (without a retrieval session) is sufficient to increase intrinsic excitability up to 24 h following fear conditioning. In addition, the increase in excitability is no longer observed despite the use of a retrieval session at 10 days post-conditioning. We cannot rule out that the enhanced excitability observed in the Cond-4d group is a result of retrieval and not the conditioning session—an important limitation to our current findings. Therefore, we conclude that even though retrieval sessions may increase excitability up to 4 days post conditioning, intrinsic excitability is no longer enhanced 10 days post-conditioning, and retrieval sessions at this time point do not increase neuronal excitability. Encoding as well as retrieval of an auditory fear memory allows linking of a subsequently formed auditory fear memory to the orginal fear memory indicating that the retrieval event is sufficient to reengage plasticty mechanisms necessary for this process (Rashid et al., 2016). Our data indicate that any behavioral effects mediated by increased excitability following memory retrieval as described in previous studies (Rashid et al., 2016; Pignatelli et al., 2019) are also transient.

### 3.2. Immediate shock enhances intrinsic excitability

To control for the effect of US presentations on LA intrinsic excitability, we used an immediate shock deficit paradigm. The phenomenon of immediate shock deficit is based on the principle that if a rodent is shocked immediately after being placed into the chamber (e.g., within 6 s), the rat displays no fear of context on a subsequent day as there was no time for a context-shock association (Fanselow, 1986). It is possible to rescue this immediate shock deficit by pre-exposure to the context the day before shock presentation (Fanselow, 1990). This allows the rats to form a context representation, indicating that immediate shock deficit is a context processing deficit (but see Lattal and Abel, 2001; Landeira-Fernandez et al., 2006). In the current study, rats were presented with the same duration of footshock as other conditioned groups (10s) immediately upon being placed in the conditioning chamber and removed from the chamber as soon as the shock presentation terminated.

We found that IS rats displayed little freezing upon exposure to the conditioning context the following day. Surprisingly, LA neurons from the IS rats did show a robust increase in neuronal excitability. The lack of any other neurophysiological data using an IS group restricts our interpretation of these data. First, it is possible that our observed changes in neuronal excitability within LA neurons are due to shock presentation rather than learning itself. We think this is unlikely as the Poor learners (animals that received conditioning but displayed little or no fear to the tone the following day) display no change in excitability relative to LA neurons from naïve rats. This demonstrates that learning, irrespective of stimulus presentation, is necessary for increased excitability.

Furthermore, repeated footshock US presentations in the absence of learning are a rodent model for stress (Valenti et al., 2011) that can result in plasticity within LA neurons. Indeed, chronic but not acute stress leads to enhanced excitability of LA neurons (Rosenkranz et al., 2010). However, it is unlikely that our observed conditioning-related changes in LA neuronal excitability result from a stress response for several reasons. Most important among these are that a single session of fear conditioning or immediate shock presentations is akin to acute stress which does not lead to changes in LA excitability. Additionally, the degree of intrinsic plasticity following chronic stress (reduced RMP, increased input resistance, reduced sAHP and spike-frequency adaptation) was more extensive than that seen in the current study.

The second explanation for excitability changes in the IS animals is that even though IS rats do not associate conditioning context with shock presentation, animals do learn to associate other cues (e.g., transport, handler, etc.) with the shock (Rudy et al., 2002). It is possible that such context learning is driving our observed changes in neuronal excitability. The most popular control for US presentation, namely pseudo-conditioning (or explicitly unpaired CS and US presentations) leads to inhibitory conditioning to the CS, i.e., animals receiving unpaired CS and US presentations encode CS as a safety signal (Rescorla and Lolordo, 1965) and display distinct forms of plasticity within amygdala (Amano et al., 2010). In addition, unpaired animals show higher context fear than those receiving paired CS-US presentations (Phillips and LeDoux, 1994). These factors make pseudo-conditioning a poor control for studies investigating learning-related neurophysiological changes in amygdala. Together, US-alone presentations (context conditioning), pseudo-conditioning as well as immediate shock paradigm make for inadequate controls to establish the effect of US presentation in the absence of learning on amygdala neurophysiology. We believe that an extended investigation of the behavioral correlates as well as neurophysiological changes in the IS group is an important area of future research. It is likely that changes in excitatory (Figure 3) and inhibitory transmission (Saar et al., 2012; Kfir et al., 2014) together with excitability changes sculpt lateral amygdala microcircuits in distinct ways following fear conditioning and immediate shock.

### 3.3. Intrinsic plasticity following fear conditioning is learning specific

The long-delay fear conditioning paradigm used here produces robust fear learning. However, we found that a few rats tested at different time points (2 for Cond-24 h, 1 each of Cond-4d and Cond-10d) displayed low levels of fear to the CS presentation indicating poor retention of memory. We found that these same rats also failed to modulate LA intrinsic excitability. It is possible that these rats had much lower basal levels of LA neuronal intrinsic excitability preventing the successful acquisition of fear learning. Several studies have demonstrated that learning performance and learning-induced changes in intrinsic excitability are correlated (Cohen-Matsliah et al., 2009; Song et al., 2012). Animals that fail to learn in trace fear conditioning or olfactory discrimination task fail to modulate neuronal excitability in the hippocampus and piriform cortex, respectively, (Cohen-Matsliah et al., 2009; Song et al., 2012). Aberrant intrinsic plasticity may also underlie aging-related learning deficits where aging animals that fail to acquire a task also fail to modulate neuronal intrinsic excitability (Moyer et al., 2000; Kaczorowski et al., 2012). Regardless, we found that intrinsic excitability levels are a good index of learning making modulation of intrinsic excitability a good target for therapeutic interventions.

### 3.4. Synaptic plasticity following fear conditioning

It is well documented that memory formation during auditory fear conditioning involves synaptic plasticity within LA neurons (McKernan and Shinnick-Gallagher, 1997; Clem and Huganir, 2010). Paired pulse ratio (PPR) is one such way to measure experience-dependent synaptic plasticity. Although previously considered a form of short-term presynaptic plasticity that reflects changes in release probability, myriad other cellular mechanisms can influence PPR (Glasgow et al., 2019). In LA neurons from naïve animals, a short interstimulus interval (ISI; e.g., 25 ms) usually results in paired-pulse facilitation (McKernan and Shinnick-Gallagher, 1997). As the ISI between pairs of presynaptic stimulation increases, the PPR is reduced. We demonstrate that long-delay fear conditioning is accompanied by a reduction in paired pulse ratio immediately as well as 24 h following fear conditioning (Figure 3). These changes were no longer evident 4 days following fear conditioning and PPR was back to naïve levels at this time point. These data are in accordance with other reports that fear learning is accompanied by a reduction in PPR at the synapses carrying auditory information onto the LA neurons.

In addition to the reduced PPR observed in fear conditioned rats, LA neurons from IS animals exhibited a robust *increase* in PPR. This increase appears to be independent of the ISI as varying the ISI did not seem to affect the PPR (Figure 3A). Therefore, footshock US presentations alone results in plasticity at thalamo-amygdala synapses. As with the changes in intrinsic excitability, it is possible that these synaptic changes in the IS group could be due to the shock presentation itself or due to learning.

### 3.5. Co-localization between synaptic and intrinsic plasticity

The current study sought to measure synaptic and intrinsic plasticity following fear learning within the same neurons. We found that fear conditioning is accompanied by a reduction in PPR at the thalamo-amygdala synapses immediately as well as 24 h following fear conditioning. These changes were no longer evident 4 days later indicating the transient nature of this form of synaptic plasticity. In addition, we observed robust changes in PPR in the immediate shock deficit group, but the direction of these changes was reversed such that LA neurons from the IS group had a larger mean PPR than LA neurons from naïve rats. More importantly, in the LA neurons from conditioned animals these changes were co-localized with the intrinsic excitability changes immediately following fear conditioning. These data support our hypothesis that intrinsic and synaptic plasticity is colocalized within lateral amygdala neurons following fear conditioning.

To investigate whether there is a relationship between intrinsic and synaptic plasticity, we looked to see whether these measures were correlated with each other. We found that intrinsic excitability and PPR were negatively correlated such that a lower PPR was associated with increased intrinsic excitability (Figure 4). In other words, synaptic changes that emerged following fear conditioning predicted intrinsic excitability changes. Specifically, neurons exhibiting the largest reduction in PPR immediately following conditioning were also the most excitable (Figure 5). This demonstrates that synaptic and intrinsic excitability following fear conditioning are co-localized to the same neurons. Interestingly, although both synaptic and intrinsic plasticity were significantly altered 24-h post conditioning, a significant correlation between these forms of plasticity was not observed at this time point. These data indicate a time-dependent shift in the relationship between intrinsic and synaptic plasticity within LA neurons following fear conditioning.

We have previously demonstrated that fear learning results in increased intrinsic excitability in ~32% of lateral amygdala neurons (Sehgal et al., 2014). These estimates are in agreement with the estimates of the proportion of lateral amygdala neurons recruited during fear conditioning (Rumpel et al., 2005; Han et al., 2007, 2009; Reijmers et al., 2007). In recent years, various genetic strategies have allowed a more precise understanding of electrophysiological changes in recently activated neurons during learning and memory formation (Cai et al., 2016; Crestani et al., 2019; Pignatelli et al., 2019; Chen et al., 2023). The use of similar genetic strategies would allow us to target neurons recruited into memory formation more precisely in future studies. However, there are some challenges to adapting these methods for our current experiments, such as the feasibility of using viral vectors in rats and the fact that neuronal labeling takes hours and therefore does not allow access to the neurons immediately after the learning event (e.g., Cond-1 h group). In addition, any particular labeling strategy (for example, those mediated by cFos, Arc, or Npas-4 expression) might not allow access to the complete neuronal ensemble underlying a particular memory (Sun et al., 2020) resulting in inaccurate conclusions. Nonetheless, the use of such genetic strategies will greatly accelerate our understanding of plasticity within neuronal ensembles underlying learning in the future.

Taken together, the current experiments demonstrate that intrinsic and synaptic plasticity in the amygdala following fear conditioning are transient and can be colocalized to the same neurons. Based on the current models of memory allocation, these data predict that future amygdala-dependent memories should be colocalized to the same neuron undergoing intrinsic plasticity albeit in a time-dependent manner.

## 4. Materials and methods

### 4.1. Subjects and behavioral training

Adult male Sprague Dawley rats (~3 months) were individually housed in clear plastic cages. Rats were maintained on a 14 h light/10 h dark cycle (lights on at 7 a.m.) with unlimited access to both water and standard laboratory rat chow (Harlan Laboratories). All procedures were conducted in accordance with the University of Wisconsin-Milwaukee animal care and use committee (ACUC) and NIH guidelines.

### 4.2. Apparatus

#### 4.2.1. Fear conditioning chambers

Fear conditioning was conducted in an apparatus previously described (Song et al., 2012; Sehgal et al., 2014). Briefly, Plexiglas and stainless steel chambers (30.5 × 25.4 × 30.5 cm; Coulbourn Instruments, Whitehall, PA) with a standard grid floor consisting of 26 parallel steel rods (5 mm diameter and 6 mm spacing) and located in a sound-attenuating box were used. The floor of the chamber was connected to a precision adjustable shock generator (Coulbourn Instruments) for delivery of a scrambled footshock, the unconditioned stimulus (US). A ventilation fan produced a constant background noise of about 50–56 dB (measured by a sound level meter, Realistic, A scale; model #33-2050, Fort Worth, TX) inside the sound attenuating box. The chamber was illuminated by a miniature incandescent white lamp (28 V, type 1819) and was wiped with a 5% ammonium hydroxide solution prior to each training session to provide a distinct olfactory cue. During training, the room lights were left on (illumination 20.9 lux) for the entire session.

#### 4.2.2. Testing chambers

An additional Plexiglas chamber served as a novel context for the auditory cue test. This chamber was located within a separate sound-attenuating box located in the same room. The test chamber was physically different from the training chamber in that it was a hexagonal chamber, the floor was black-painted Plexiglas, and it was illuminated with an infrared light. In addition, the tray below the test chamber floor contained clean bedding and the test chamber was wiped with 2% acetic acid prior to each test session to provide a different olfactory stimulus from that used during training. The room lights were turned off (illumination 0.2 lux) for the entire testing session.

### 4.3. Behavioral groups

#### 4.3.1. Training

Rats were handled for 7 days and habituated to transportation for 3 days. After handling and habituation, rats were randomly divided into seven groups (Figure 1A). On day 0, the 4 experimental groups received one 10-trial session of auditory long-delay fear conditioning using a 45 s CS (conditioned stimulus, 80 dB white noise) followed by a brief footshock US (unconditioned stimulus; 1 s; 1 mA), and a 5.2 min intertrial interval (ITI). We have previously demonstrated that this long-delay fear conditioning protocol results in robust freezing in response to the CS but low levels of freezing to the training context (Detert et al., 2008). Thus, this particular fear conditioning protocol allows us to determine the effect of auditory fear learning on lateral amygdala intrinsic plasticity while minimizing any confounding effects of context fear conditioning. To determine the time course of fear conditioning-related intrinsic plasticity, brain slices from the Cond-1 h, Cond-24 h, Cond-4d, and Cond-10d rats were obtained either 1 h, 24 h, 4d, or 10d following fear conditioning, respectively. The three Control groups were experimentally naive rats (never handled, Naive), or rats that received unpaired CS (CS-alone) or US (US-alone) trials. The CS-alone group received 10 CS presentations separated by a 5.2 min ITI on day 0 and served as a control for chamber exposure as well as CS presentation. The CS-alone group spent the same amount of time in the conditioning chamber as the conditioned animals but did not receive the US. The rats from the US-alone (immediate shock) group were placed in the training chamber where they received 10 continuous US presentations (1 s, 1 mA each) immediately afterwards and were rapidly removed from the chamber. Such US presentations in the absence of context exploration or encoding lead to a failure to associate the training chamber with US presentations and has been described as immediate shock deficit paradigm (Fanselow, 1986). This US-alone control group allows us to determine any effect US presentations alone (in the absence of associative fear conditioning) might have on LA intrinsic excitability. The US-alone control group was placed in the conditioning chamber for 10 s and quickly removed.

To assess memory retention, rats in the CS-alone, IS, Cond-24 h, Cond-4d, and Cond-10d group were given the same probe test in a novel context immediately before slice preparation. Any conditioned animals that displayed less than 50% freezing during CS presentation were separated into a *Poor learners* group. The immediate shock group was tested in the conditioning chamber (instead of a novel chamber) to assess context-shock association.

#### 4.3.2. Analyses of behavioral data

The training sessions were recorded using a remote CCTV video camera (model #WV-BP334; Panasonic Corp., China) mounted to the top of each behavioral chamber. The video data were fed to a PC running FreezeFrame 2.04. Freezing was defined as the absence of all movement except that required for respiration (Blanchard and Blanchard, 1969) and a 1 s bout of immobility was scored as freezing using FreezeView 2.04 (Actimetrics Software, Coulbourn Instruments).

### 4.4. Slice preparation

Brain slices were prepared by an individual blind to the training condition. Rats were deeply anesthetized with isoflurane, perfused with ice-cold oxygenated sucrose aCSF (composition in mM: 2.8 KCl, 1.25 NaH_2_PO_4_, 2 MgSO_4_, 1 CaCl_2_, 1 MgCl_2_ 26 NaHCO_3_, 248 Sucrose and 10 dextrose) and decapitated. The brain was quickly removed and placed in ice-cold oxygenated (95% O_2_/5% CO_2_) aCSF (composition in mM: 124 NaCl, 2.8 KCl, 1.25 NaH_2_PO_4_, 2 MgSO_4_, 2 CaCl_2_, 26 NaHCO_3_, and 20 dextrose). The brain was then blocked and 400 μm-thick coronal brain slices were cut in aCSF at ~0°C using a vibrating microtome (VT1200, Leica). Only slices that were located between 1.88 and 3.30 mm posterior to bregma, thus containing lateral amygdala, were used (Paxinos and Watson, 1998). Slices were then transferred to a holding chamber (Moyer and Brown, 1998) containing oxygenated aCSF at 32–36°C and allowed to recover for 30 min. The slices were then kept at room temperature (21–23°C) for an additional 30 min before any electrophysiological recordings began.

### 4.5. Electrophysiological recordings

For electrophysiological measurements, slices were transferred as needed to the recording chamber, where they were perfused with oxygenated aCSF at 32°C. The slices were held in place using nylon strands stretched across a U-shaped platinum wire. Visually-guided whole cell patch clamp recordings were made using infrared differential interference contrast optics. All recordings were obtained using a MultiClamp 700B amplifier system (Molecular Devices, Union City, CA). Experiments were controlled by PClamp 10 software running on a PC, and the data were acquired using the Digidata 1440A acquisition system. All recording electrodes (3–8 MΩ) were pulled from thin-walled capillary glass (A-M Systems, Carlsborg, WA) using a Sutter Instruments P97 puller. The patch pipettes were filled with internal solution containing (in mM) 110 K-gluconate, 20 KCl, 10 Di-Tris-P-Creatine, 10 HEPES, 2 MgCl_2_, 2 Na_2_ATP, 0.3 Na_2_GTP, 0.2% Biocytin, with a pH of 7.3 and osmolarity of 290 mOsm. Only cells with a stable, uncorrected resting membrane potential (RMP) between −50 and −85 mV, overshooting action potentials, and an input resistance (R*_N_*) >100 MΩ were used. Figure 2A shows a photograph of a coronal slice containing lateral amygdala with the typical recording site noted.

#### 4.5.1. Intrinsic excitability

To minimize the influence of voltage-dependent changes on membrane conductances, all cells were studied at a membrane potential near −60 mV (using constant current injection under current clamp mode). To study intrinsic excitability, WCRs were conducted under current clamp using the following protocol:

1. Voltage–current (V-I) relations were obtained using 400 ms current steps (range −50 pA to rheobase) and by plotting the plateau voltage deflection against current amplitude. Neuronal input resistance (R*_N_*) was determined from the slope of the linear fit of that portion of the V-I plot where the voltage sweeps did not exhibit sags or active conductance.
2. Intrinsic excitability measurements were obtained using 1 s current steps (range 0–500 pA) and by plotting the number of action potentials fired against current amplitude.
3. Spike-frequency adaptation (accommodation; 3X, at 30 s intervals) was studied using a 1 s depolarizing current injection of the same stimulus intensity that was just sufficient to elicit a burst of three action potentials using a 100 ms depolarizing current injection. For each sweep, the number of action potentials elicited were counted.
4. Resting membrane potential (RMP) was calculated as the difference between mean membrane potential during the first minute immediately after obtaining whole cell configuration and after withdrawing the electrode from the neuron.

#### 4.5.2. Synaptic plasticity measurements

In a subset of experiments after obtaining measures of intrinsic excitability under current-clamp mode, measures of synaptic plasticity were obtained. All measurements were performed in ACSF containing picrotoxin (100 μm) to obtain pure excitatory responses.

1. Under voltage-clamp configuration, baseline synaptic transmission was measured by obtaining an input-output (I/O) curve. I/O curves were generated by presynaptic stimulation (100 μs duration) to the thalamic fibers traversing the LA using a concentric bipolar microelectrode (FHC, Brunswick, ME). Varying stimulation intensities (from EPSC threshold to EPSC spike generation) were plotted against the resultant peak EPSC amplitude and EPSC slope. To ensure that evoked excitatory postsynaptic currents (EPSCs) were monosynaptic the following criteria were used: (a) the latency of the response, on average 3–4 ms (Clem and Huganir, 2010), (b) response latencies remained constant across stimuli (Weisskopf et al., 1999; Clem and Huganir, 2010), (c) only the early part the EPSC/EPSP slope was measured (Bauer et al., 2002), and (d) only the lowest stimulation intensities were used, unless otherwise noted (Weisskopf et al., 1999).
2. Paired pulse ratios (PPR) were obtained to determine any changes in presynaptic release probabilities. Pairs of EPSCs were generated by two temporally close presynaptic stimulations ranging from 25 to 150-ms apart at 0.1 Hz. Stimulation intensities were kept to a minimum such that these generated a reliable EPSC (greater than 50 pA) without recruiting polysynaptic responses or spiking. PPR were calculated as the ratio of the peak of the second EPSC to the first EPSC (McKernan and Shinnick-Gallagher, 1997).

#### 4.5.3. Characterization of LA pyramidal neurons

We used electrophysiological criteria to confirm that all electrophysiological recordings were obtained from LA pyramidal neurons (spike frequency adaptation, input resistance, spike half width and fast AHP). For example, most LA interneurons display very little spike frequency adaptation and fire at high frequencies following large somatic depolarization (~100 Hz). Data from LA interneurons were excluded from the analysis.

### 4.6. Biocytin staining

Neurons were filled with biocytin to confirm the position and identity of pyramidal cells in LA. After obtaining electrophysiological measurements, slices were fixed in 10% neutral-buffered formalin at 4°C for 1 to 7 days before further processing. To visualize LA neurons, slices were incubated in 3% H_2_O_2_/10% methanol for 45 min, washed with PBS for 10 min (3X), followed by 0.25% Triton X-100/ 2% BSA for 60 min. The slices were then incubated with 1:500 streptavidin Alexa Fluor 488 (Invitrogen) for 120 min in the dark and washed with PBS for 10 min (3X). They were mounted onto slides, coverslipped with Ultra Cruz Mounting Medium (Santa Cruz Biotechnology, Santa Cruz, CA), and sealed with nail polish. The neurons were viewed and photographed using a fluorescence microscope (BX51WI, Olympus) at 20X or Olympus Fluoview FV1200 confocal system. Neurons were classified as pyramidal when either a prominent apical dendrite, large soma or dendritic spines were detected. Representative biocytin-filled LA pyramidal neurons are shown in Figure 2A.

### 4.7. Statistical analyses

The main effects and *post hoc* comparisons were analyzed using ANOVA. Additionally, Pearson’s and Spearman’s correlation coefficients were calculated to determine whether post-training intrinsic excitability was correlated with post-training synaptic plasticity. All data were expressed as mean ± SEM. All statistical analyses were performed on cells.

## Data availability statement

The original contributions presented in the study are included in the article/supplementary material, further inquiries can be directed to the corresponding author.

## Ethics statement

The animal study was approved by University of Wisconsin-Milwaukee animal care and use committee (ACUC). The study was conducted in accordance with the local legislation and institutional requirements.

## Author contributions

MS and JM did experimental design, drafted and revised the manuscript. MS performed the electrophysiological experiments. VE helped with the behavioral training. All authors contributed to the article and approved the submitted version.
